# A converging reputation ranking iteration method via the eigenvector

**DOI:** 10.1371/journal.pone.0274567

**Published:** 2022-10-03

**Authors:** Xiao-Lu Liu, Chong Zhao

**Affiliations:** 1 School of Management Science and Engineering, Shandong University of Finance and Economics, Jinan, PR China; 2 School of Mathematics, Shandong University, Jinan, PR China; National Institute of Advanced Industrial Science and Technology, JAPAN

## Abstract

Ranking user reputation and object quality in online rating systems is of great significance for the construction of reputation systems. In this paper we put forward an iterative algorithm for ranking reputation and quality in terms of eigenvector, named EigenRank algorithm, where the user reputation and object quality interact and the user reputation converges to the eigenvector associated to the greatest eigenvalue of a certain matrix. In addition, we prove the convergence of EigenRank algorithm, and analyse the speed of convergence. Meanwhile, the experimental results for the synthetic networks show that the AUC values and Kendall’s *τ* of the EigenRank algorithm are greater than the ones from the IBeta method and Vote Aggregation method with different proportions of random/malicious ratings. The results for the empirical networks show that the EigenRank algorithm performs better in accuracy and robustness compared to the IBeta method and Vote Aggregation method in the random and malicious rating attack cases. This work provides an expectable ranking algorithm for the online user reputation identification.

## Introduction

User reputation measures the user ability of rating accurately to various objects. In the interpretation of online rating systems, one of the central problems is how to construct the personal reputation systems [1–3], which could have an impact on e-commerce [4, 5], recommender systems [6, 7], rumor spreading [8, 9], etc. In the past several years, online rating systems of many platforms provide user channels to express their preferences to different objects. Nevertheless, not every user gives reasonable/accurate ratings due to his/her dishonesty or non-familiarity [2, 10]. Inaccurate ratings affect the normal sales of online businesses, endangering the healthy development of the social economy. Therefore, measuring the online user reputation according to their rating behaviors is crucial for the maintenance of a good market order and construction of strong cyberpower [11–14].

In the existing works, the iteration-oriented mechanisms have been widely explored, Pagerank [15] and HITS algorithms [16] as representatives. In recent years, quality-based ranking methods are introduced, in which each object is assumed to have an inherent quality and the user reputation is characterized by the relations between his/her rating vector and the corresponding objects’ calculated quality vector. User reputation and object quality are interdependent and are updated iteratively until they become stable. Laureti *et al*. [17] raised an iterative refinement (abbr. IR) method by analyzing the difference between user rating and object quality vectors. Similarly, there is improved IR method [18]. Zhou *et al*. [19] proposed a correlation-based ranking (abbr. CR) method in terms of Pearson correlation coefficient between vectors of user rating and object quality. Similarly, there are IARR method [20], IRUA method [21], etc. [22]. Besides, Allahbakhsh *et al*. [23] proposed a Vote Aggregation method by computing the grade of credibility, and assigning each user a degree of consistence with the community sentiment. Along this line, there are group-based ranking method [24] and so on [25–27]. In addition, Liu *et al*. [28] designed a reputation ranking algorithm via the beta distribution (RBPD for short), in which the user reputation is determined by the probability that the user is to rate fairly. And IBeta method [29] is proposed by introducing an iterative reputation-allocation process based on the RBPD method.

Among the existing reputation ranking methods, the iterative algorithms could measure the user reputation in good accuracy. However, many iterative methods do not converge, or omit the proof of theoretical convergence [19, 20, 29]. It is expected that iterative algorithm with definite convergence can lead to more robust reputation lists [26, 30]. Inspired by this thought, in this paper we develop an iterative reputation and quality ranking algorithm based on eigenvector analysis, called the EigenRank algorithm, where the user reputation and object quality are interdependent and the user reputation vector series converges to the eigenvector corresponding to the largest eigenvalue of a certain matrix. In addition, we give the proof of the convergence of EigenRank algorithm, and analyse the speed of convergence. Meanwhile, we explore the ranking performance of the EigenRank algorithm for the synthetic and empirical networks compared with the IBeta method and Vote Aggregation method. Our results for the synthetic networks imply that the AUC values and Kendall’s *τ* of the EigenRank algorithm exceed their counterparts obtained from the IBeta method and Vote Aggregation method with different proportions of random/malicious ratings. The results for the empirical networks show that the EigenRank algorithm has a better performance in accuracy and robustness than the IBeta method and Vote Aggregation method in the random and malicious rating attack cases.

We organize this paper as follows. The EigenRank algorithm is presented in section II. In sections III and IV, we show the experimental results for the synthetic networks and empirical networks, respectively. Finally, section V gives the conclusion and discussions. The analysis of convergence for the EigenRank algorithm is put in the Supporting Information.

## The EigenRank algorithm

We use a weighted bipartite network *G* = {*U*, *O*, *E*} to represent the rating system, where *U* is the user set, *O* is the object set and *E* is the rating set. For the sake of clarity, Latin and Greek letters are used to represent attributes of users and objects, respectively. We use *r*_*iα*_ to denote the rating given by user *i* to object *α*, and list the ratings into a matrix **A**, called the rating matrix. It is noted that, in the case the range of ratings varies with object, the ratings should be linearly normalized into a fixed interval, for instance [0, 1], before constructing the matrix **A**. The letters used in this paper are shown in Table 1.

**Table 1:** Descriptor table of letters used in this paper.

Letters	Descriptor
*r*_*iα*_	the rating given by user *i* to object *α*
*d*_*i*_	degree of user *i*
*k*_*α*_	degree of object *α*
**A**	the rating matrix
**B**	the normalized rating matrix
**R**	user reputation vector
**Q**	object quality vector
**D**	diag{*d*_1_, *d*_2_, …, *d*_|*U*|_}
**K**	diag{*k*_1_, *k*_2_, …, *k*_|*O*|_}

Firstly, we normalize the ratings as follows. Because different users have different rating criteria, part of them tend to give high ratings and others give low ones, therefore ratings do not reflect the real opinion of the users to objects precisely. To balance this deviation, we need to make some normalization, the rating *r*_*iα*_ is transformed to the extent of fanciness $r_{i\alpha }^{\prime }$,

$$\begin{array}{c} r_{i\alpha }^{\prime }=\{\begin{array}{cc} 2\left(r_{i\alpha }-r_{i}^{2}\right)/\left(r_{i}^{1}-r_{i}^{2}\right)-1, & r_{i}^{1}\neq r_{i}^{2}\\ 0, & r_{i}^{1}=r_{i}^{2} \end{array}, \end{array}$$ (1)

where $r_{i}^{1}$ and $r_{i}^{2}$ are the maximum and minimum ratings user *i* gives, respectively. Thus, all the ratings given by a specific user are normalized to [-1, 1], so that the extreme ratings are set 1 and -1, respectively. Particularly, the normalized ratings are all 0 if and only if the user gives constant rating. Positive $r_{i\alpha }^{\prime }$ reflects positive opinion and negative $r_{i\alpha }^{\prime }$ represents negative opinion. The normalized rating are arranged in a matrix **B**, whose (*i*, *α*)-th entry is $r_{i\alpha }^{\prime }$.

Initially, the user reputation **R**^(0)^ is assigned in terms of the user degree, *R*_*i*_ = *k*_*i*_/|*O*| (where |*O*| is the number of objects). Object quality is proportional to the received average normalized rating, weighted by user reputation.

We tempt to express the object quality as

$$\begin{array}{c} Q=c_{1}f\left(K\right)B^{T}R, \end{array}$$ (2)

where *f* is a continuous monotone function on [0, ∞), and *c*_1_ > 0 is an adjustable parameter that does not influence the relative rank of object quality **Q**. To determine this function *f*, imagine that if each user has a brother, whom gives the same rating as *i* did to each object. In this case, the relative rank of the objects should not be disturbed, and the rating matrix becomes $\left(\begin{array}{c} B\\ B \end{array}\right)$ instead of **B**, and **R** is replaced by $\left(\begin{array}{c} R\\ R \end{array}\right)$. Then formula (2) becomes

$$\begin{array}{c} Q=c_{1}^{\prime }f\left(2K\right)B^{T}R, \end{array}$$ (3)

for some new constant $c_{1}^{\prime }$. Comparing (3) with (2) conclude

$$\begin{array}{c} f\left(2k_{\alpha }\right)=cf\left(k_{\alpha }\right),\forall \alpha \in O \end{array}$$ (4)

for some constant *c* independant on *α*. Suppose $c=2^{u_{1}}$, then (4) can be reformed as

$$\begin{array}{c} {\left(2k_{\alpha }\right)}^{-u_{1}}f\left(2k_{\alpha }\right)=k_{\alpha }^{-u_{1}}f\left(k_{\alpha }\right),\forall \alpha \in O. \end{array}$$ (5)

Suppose *f* satisfies ${\left(2x\right)}^{-u_{1}}f\left(2x\right)=x^{-u_{1}}f\left(x\right),x\in \left[0,\infty \right)$ and $v={\text{lim}}_{x\to 0+}x^{-u_{1}}f\left(x\right)$ exists, then for each *x* > 0 we have $x^{-u_{1}}f\left(x\right)={\text{lim}}_{n\to \infty }{\left(2^{-l}x\right)}^{-u_{1}}f\left(2^{-l}x\right)=v$, therefore $x^{-u_{1}}f\left(x\right)\equiv v$, i.e. $f\left(x\right)=vx^{u_{1}},\forall x$. Consequently, the object quality can be expressed as

$$\begin{array}{c} Q=c_{1}K^{u_{1}}B^{T}R. \end{array}$$ (6)

User reputation is measured by the relationship between the user’s normalized rating and object quality. When users have a positive view to the object, the larger is the object quality, the higher is the user reputation; Conversely, when the user has a negative view of the object, the smaller is the object quality, the higher is the user reputation. From this viewpoint, the user reputation can be expressed by

$$\begin{array}{c} R=c_{2}D^{u_{2}}BQ, \end{array}$$ (7)

where *c*_2_, *u*_2_ are adjustable parameters, and *c*_2_ > 0 does not change the relative rank of user reputation **R**.

When *u*_2_ = 0, the opinions of inactive users will be covered up by those of active ones; meanwhile, When *u*_2_ = −1, the opinions of active and inactive users have the same weight. These two choices are too extreme, hence we make a compromised choice, $u_{2}=-\frac{1}{2}$ in our algorithm. Similarly, *u*_1_ is also set $-\frac{1}{2}$.

At each step, the vectors **R** and **Q** will be updated according to formulae (6) and (7). The reputation vector after the *n*-th iteration step is recorded as **R**^(*n*)^. As *n* → ∞, **R**^(*n*)^ converges to the unit eigenvector associated to the greatest eigenvalue of a certain matrix, and we put its proof in the S1 Appendix. The iteration process will not stop until the reputation vector becomes stable, i.e. the distance $\left|\right|R^{\left(n\right)}-R^{\left(n-1\right)}{\left|\right|}_{2}^{2}$ between the reputation vectors decays to below the threshold *δ* = 10^−10^, where

$$\begin{array}{c} \left|\right|R^{\left(n\right)}-R^{\left(n-1\right)}{\left|\right|}_{2}^{2}=\frac{1}{\left|U\right|}\sum_{i\in U}{\left(R_{i}^{\left(n\right)}-R_{i}^{\left(n-1\right)}\right)}^{2}. \end{array}$$ (8)

The numerical convergence of EigenRank algorithm is theoretically guaranteed, of which the proof is put in the S1 Appendix. We discuss the parameters *c*_1_ and *c*_2_. Substituting formula (6) into formula (7), we get

$$\begin{array}{c} R=c_{1}c_{2}D^{-\frac{1}{2}}BK^{-\frac{1}{2}}B^{T}R. \end{array}$$ (9)

One can find that **R** is an eigenvector associated to the largest eigenvalue of the matrix $D^{-\frac{1}{2}}BK^{-\frac{1}{2}}B^{T}$. Let λ_*m*_ denote the largest eigenvalue of matrix $D^{-\frac{1}{2}}BK^{-\frac{1}{2}}B^{T}$, then $D^{-\frac{1}{2}}BK^{-\frac{1}{2}}B^{T}R=\lambda_{m}R$, we find that the parameters *c*_1_ and *c*_2_ satisfy,

$$\begin{array}{c} c_{1}c_{2}=\frac{1}{\lambda_{m}}. \end{array}$$ (10)

As mentioned before, the values of *c*_1_, *c*_2_ do not influence the relative rank of reputation **R**. In fact, what we need is the direction of the vector **R**, which determines the relative ratios of its coordinates, rather than its length. In each step of iteration, we normalize **R**^(*n*)^ in the 2-norm, which do not impact on its direction, and could make the sequence **R**^(*n*)^ converge. The procedure of EigenRank Algorithm is summarized in Algorithm 1.

The convergence analysis of EigenRank algorithm can be seen in the S1 Appendix. We find that the iteration stops when the number of steps $n>{\text{log}}_{\eta }\frac{x_{1}^{\left(0\right)}\delta }{16||D^{-\frac{1}{4}}\left|\right|\cdot \left|\right|D^{\frac{1}{4}}\left|\right|\cdot \left|\right|x^{\left(0\right)}{\left|\right|}_{2}}+1$. In addition, the total complexity of the EigenRank algorithm is *O*(|*U*||*O*|log_*η*_ *δ*).

**Algorithm 1** The iterative EigenRank Algorithm

**Input**: Rating matrix **A**;

**Output**: User reputation matrix **R** and object quality matrix **Q**;

1: Make normalization of ratings;

2: Initialize **R**^(0)^ in terms of user degree;

3: **while** $\left|\right|R^{\left(n\right)}-R^{\left(n-1\right)}{\left|\right|}_{2}^{2}<\delta =10^{-10}$ *is not met* **do**

4:  $Q\leftarrow K^{-\frac{1}{2}}B^{T}R$;

5:  $R\leftarrow D^{-\frac{1}{2}}BQ/\left|\right|D^{-\frac{1}{2}}BQ{\left|\right|}_{2}$;

6: **end**

7: **return** **R** and **Q**

## Results for synthetic networks

Moreover, we analyze the ranking performance of the EigenRank algorithm compared to the IBeta method and Vote Aggregation method for the synthetic networks, in which we firstly add the weighted links by employing the preferential attachment mechanism [31] and then insert different number of distorted ratings.

When we generate the synthetic networks, the numbers of users and objects are set |*U*| = 6000 and |*O*| = 6000, respectively. The network sparsity *η* is set to 0.02, 0.03, respectively. We add the weighted links (ratings) one after one until the total cardinality of the ratings |*E*| reach *η* × |*U*||*O*| = 7.2 × 10^5^, 1.08 × 10^6^, respectively. The nodes (user and object) of links are selected in terms of the node degree preferentially. The possibility of selecting user *i* and object *α* at each time step *t* are formulated as

$$\begin{array}{c} p_{i}\left(t\right)=\frac{k_{i}\left(t\right)+1}{\Sigma_{j\in U}\left(k_{j}\left(t\right)+1\right)}, \end{array}$$ (11)

$$\begin{array}{c} p_{\alpha }\left(t\right)=\frac{k_{\alpha }\left(t\right)+1}{\Sigma_{\beta \in O}\left(k_{\beta }\left(t\right)+1\right)}, \end{array}$$ (12)

where *k*_*i*_(*t*) is the degree of user *i* at the time step *t* and *k*_*α*_(*t*) is the degree of object *α* at the time step *t*.

Two ingredients contribute to the rating *r*_*iα*_, the link weight from user *i* to object *α*: the object inherent quality $Q_{\alpha }^{\prime }$, and the rating error Δ*δ*_*iα*_. Here $Q_{\alpha }^{\prime }$ subjects to uniform distribution *U*(1, 5) and Δ*δ*_*iα*_ is drawn from the normal distribution $N(0,\Delta \delta_{i}^{2})$, in which Δ*δ*_*i*_ is the rating error of user *i*, subjecting to *U*(1, 5). The rating *r*_*iα*_ is calculated by

$$\begin{array}{c} r_{i\alpha }=\left[Q_{\alpha }^{\prime }+\Delta \delta_{i\alpha }\right], \end{array}$$ (13)

where [ ] represents the closest integer to $Q_{\alpha }^{\prime }+\Delta \delta_{i\alpha }$. The rating *r*_*iα*_ is limited to the set {1, 2, 3, 4, 5}, and we will truncate the ratings beyond {1, 2, 3, 4, 5}.

Then, we replace part of the links by the distorted ratings. In the synthetic networks, we assume two kinds of distorted ratings simultaneously exist in real online rating systems, those are, random ratings and malicious ones. The former are given by users who rate objects totally randomly in the set {1, 2, 3, 4, 5}, while the latter arise when some of users always give extreme ratings to push up or down certain target objects. In the synthetic networks, we replace *ρ* proportion of the original links by the distorted ratings (random/malicious case). Larger parameter *ρ* reflects more noisy information. *ρ* = 0 represents there is all true information and *ρ* = 1 means totally chaos. In our synthetic experiments, the proportion *ρ* is set to 0.025, 0.05, …, 0.5, successively.

To characterize the ranking performance of the EigenRank algorithm, we introduce two indices: AUC curve [32] (the area under a receiver operating characteristic curve) and Kendall’s *τ* [33]. When calculating the AUC value, we need to divide the objects into two subsets, i.e. the benchmark objects and non-benchmark ones, according to their qualities. After *n* times of independent comparisons, there are *n*_1_ times that the benchmark one has a higher quality than its opponent, and *n*_2_ times they reach a draw. The AUC value is calculated by

$$\begin{array}{c} AUC_{s}=\frac{2n_{1}+n_{2}}{2n}, \end{array}$$ (14)

where the parameter *n* = 1 × 10^9^ in the experiments. When *AUC*_*s*_ = 1, the selected benchmark object has a higher rank than its opponent in all the comparisons. While *AUC*_*s*_ = 0.5 implies all the objects are randomly ranked. In the synthetic networks, we select 20% highest-quality objects as benchmark objects based on their inherent qualities $Q_{\alpha }^{\prime }$. The higher is the AUC value, the more accurate is the ranking of object quality.

Another index Kendall’s *τ* computes the rank correlation between the inherent quality *Q*′ and the obtained object quality *Q*, which is formulated by

$$\begin{array}{c} \tau =\frac{2}{\left|O|(|O|-1\right)}\sum_{\mu <\nu }sgn\left[\left(Q_{\mu }^{\prime }-Q_{\nu }^{\prime }\right)\left(Q_{\mu }-Q_{\nu }\right)\right], \end{array}$$ (15)

where *sgn*(*x*) is the sign function. $\left(Q_{\mu }^{\prime }-Q_{\nu }^{\prime }\right)\left(Q_{\mu }-Q_{\nu }\right)>0$ means *Q*′ and *Q* is concordant and negative indicates discordant. *τ* always lies in [−1, 1] and higher Kendall’s *τ* indicates higher ranking accuracy.

We compare the ranking performance of the EigenRank algorithm to IBeta method and Vote Aggregation method. Considering Vote Aggregation method has parameters, in our experiments we set the parameters the same as in the examples from [23].

**Fig 1:**
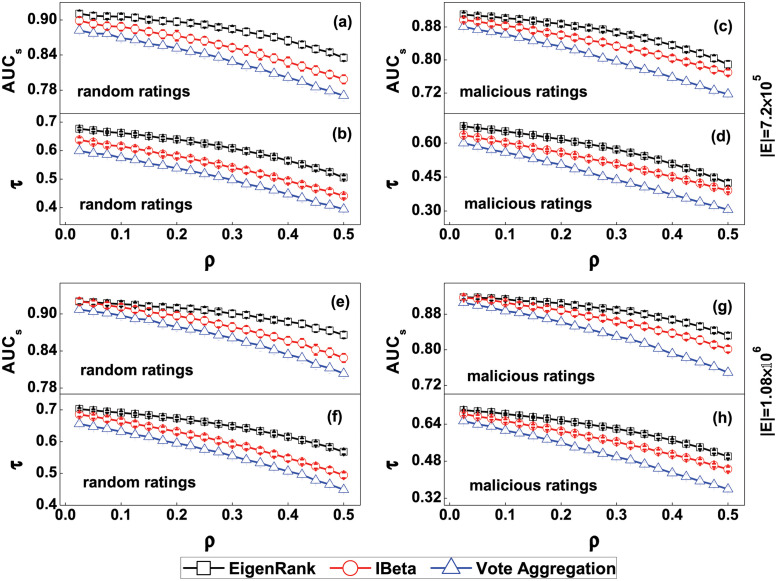
(Color online) *AUC*_*s*_ and *τ* of the EigenRank algorithm, IBeta method and Vote Aggregation method for the synthetic networks: (a, b) in the random rating attack case when |*E*| = 7.2 × 10^5^; (c, d) in the malicious rating attack case when |*E*| = 7.2 × 10^5^; (e, f) in the random rating attack case when |*E*| = 1.08 × 10^6^; (g, h) in the malicious rating attack case when |*E*| = 1.08 × 10^6^. The parameter *ρ* denotes the ratio of random/malicious ratings. It can be seen that both the *AUC*_*s*_ and *τ* of the EigenRank algorithm exceed their counterparts in the IBeta method and Vote Aggregation method with different *ρ* and |*E*|. The results are averaged over 50 independent realizations. The error bars are the corresponding standard deviations.

Fig 1 shows the AUC values *AUC*_*s*_ and Kendall’s *τ* of the EigenRank algorithm, IBeta method and Vote Aggregation method via different proportions of distorted ratings (random/malicious case) for the synthetic networks. From Fig 1(a) and 1(b) one sees that both *AUC*_*s*_ and Kendall’s *τ* of the EigenRank algorithm are larger than their counterparts in the IBeta method and Vote Aggregation method for different parameter *ρ* in the random rating attack case when |*E*| = 7.2 × 10^5^. For example, when *ρ* = 0.1, the *AUC*_*s*_ of the EigenRank algorithm, IBeta method and Vote Aggregation method could reach 0.906, 0.888 and 0.869, respectively, and the corresponding *τ* could reach 0.662, 0.615 and 0.574, respectively. From Fig 1(c) and 1(d) we observe that both *AUC*_*s*_ and *τ* of the EigenRank algorithm are greater than the ones from the IBeta method and Vote Aggregation method for different *ρ* in the malicious rating attack case when |*E*| = 7.2 × 10^5^. For instance, when *ρ* = 0.1, the *AUC*_*s*_ of the EigenRank algorithm, IBeta method and Vote Aggregation method reach 0.902, 0.883 and 0.862, respectively, and the corresponding *τ* reach 0.651, 0.602 and 0.559, respectively. One also finds that both *AUC*_*s*_ and *τ* of the EigenRank algorithm exceed their counterparts in the IBeta method and Vote Aggregation method with different *ρ*, not only in the random rating attack case Fig 1(e) and 1(f) but also in the malicious rating attack case Fig 1(g) and 1(h) when |*E*| = 1.08 × 10^6^. For example, when *ρ* = 0.2 in the random rating attack case, the *AUC*_*s*_ of the EigenRank algorithm, IBeta method and Vote Aggregation method reach 0.910, 0.897 and 0.879, respectively. When *ρ* = 0.2 in the malicious rating attack case, the *τ* of the EigenRank algorithm, IBeta method and Vote Aggregation method could reach 0.656, 0.610 and 0.561, respectively. The results imply that the EigenRank algorithm measures the user reputation and object quality more accurately than the IBeta method and Vote Aggregation method.

## Results for empirical networks

Furthermore, we explore the ranking performance of the EigenRank algorithm compared with the IBeta method and Vote Aggregation method via two empirical data sets which contain ratings given from users to movies: MovieLens and Netflix. The MovieLens data is downloaded from the GroupLens (http://www.grouplens.org), and the Netflix data is provided from the Netflix Prize (http://www.netflixprize.com). Both of two empirical data sets are extracted from the original data and have 1000209 and 824802 ratings for the MovieLens and Netflix data set, respectively. Each user has at least 20 ratings and the ratings in the two data sets belong to {1, 2, 3, 4, 5}, in which rating 5 represents liking best and rating 1 indicates liking least. Table 2 characterizes some basic statistical properties of the two data sets.

**Table 2:** Basic statistical properties of the empirical data sets used in this paper. |*U*|, |*O*| and |*E*| denote the cardinality of users, objects and ratings, respectively. 〈*k*_*U*_〉 and 〈*d*_*O*_〉 are the average degree of users and objects, respectively. *η* records the network sparsity.

Data Sets	|*U*|	|*O*|	|*E*|	〈*k*_*U*_〉	〈*d*_*O*_〉	*η*
MovieLens	6040	3706	1000209	166	270	0.0447
Netflix	10000	6000	824802	82	137	0.0137

In the empirical networks, distorted ratings (random/malicious case) are also assumed to exist, similar to the synthetic networks. The artificial spammers in two empirical networks are generated with two kinds of attacks separately: random rating attack and malicious rating attack. In both cases, we randomly select some users and assign them distorted ratings: randomly from {1, 2, 3, 4, 5} in the former case; half chance rating 1 and half chance rating 5 in the latter case. The ratio of spammers is *q* and the activity of spammers is *p*. For the empirical data sets, we randomly select *q*|*U*| users as the spammers, and then assign *p*|*O*| distorted ratings to each spammer [24]. If the degree *k*_*i*_ of a selected spammer *i* is greater than *p*|*O*|, we randomly distort his/her *p*|*O*| ratings, then truncate the remaining *k*_*i*_ − *p*|*O*| ratings. Otherwise, we distort all the spammers’ ratings, then randomly select *p*|*O*| − *k*_*i*_ of his/her non-rated objects and give them distorted ratings.

The AUC curve [32] is again used to characterize the ranking performance of the EigenRank algorithm for the empirical networks. We introduce another index, recall [34]. To calculate the AUC values, we set the spammers and non-spammers as benchmark and non-benchmark, respectively. We conduct *n*′ times independent comparisons of pairs of users: each pair consisting of a spammer and a non-spammer, chosen randomly. Denote $n_{1}^{\prime }$ the number of chances that the spammer has lower reputation than the non-spammer, and $n_{2}^{\prime }$ the number of chances that the two selected users have equal reputation. The AUC value is expressed as

$$\begin{array}{c} AUC_{e}=\frac{2n_{1}^{\prime }+n_{2}^{\prime }}{2n^{\prime }}, \end{array}$$ (16)

where the parameter *n*′ = 1 × 10^9^ in the experiments, as we did in the experiments for synthetic networks. The higher is the AUC value, the more accurate is the ranking of user reputation.

The recall measures to what extent the spammers can be actually identified in the *L*-lowest reputation users,

$$\begin{array}{c} R_{c}\left(L\right)=\frac{d^{\prime }\left(L\right)}{q|U|}, \end{array}$$ (17)

where *d*′(*L*) is the number of identified spammers in the *L*-lowest reputation users and *L* = *q*|*U*|. It’s noted that *d*′(*L*) < *q*|*U*| and *R*_*c*_(*L*) ∈ [0, 1]. Higher *R*_*c*_(*L*) indicates more accurate reputation ranking list, and vice versa.

**Fig 2:**
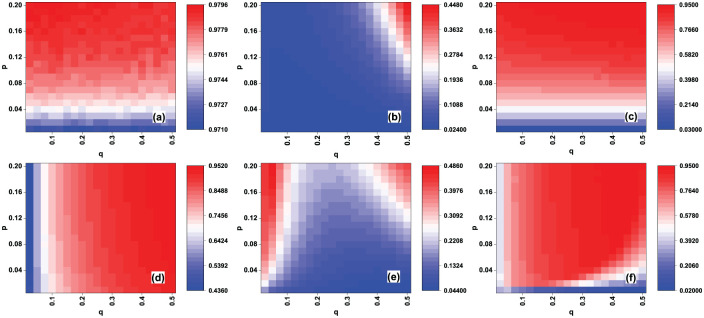
(Color online) The effectiveness of the EigenRank algorithm under random rating attack for the MovieLens data set: (a) the AUC values *AUC*_*e*_; (b-c) Δ*AUC*_*e*_, the comparison of *AUC*_*e*_ between EigenRank algorithm and IBeta method (b), Vote Aggregation method (c); (d) the recall *R*_*c*_(*L*); (e-f) Δ*R*_*c*_(*L*), the comparison of recall between EigenRank algorithm and IBeta method (e), Vote Aggregation method (f). The parameters *q* and *p* denote the ratio and the activity of spammers, respectively. *q* ranges from 0.025 to 0.5 in increments of 0.025 and *p* ranges from 0.01 to 0.2 in increments of 0.01. One observes both the *AUC*_*e*_ and *R*_*c*_(*L*) of the EigenRank algorithm exceed the ones obtained by the IBeta method and Vote Aggregation method for different (*p*, *q*).

**Fig 3:**
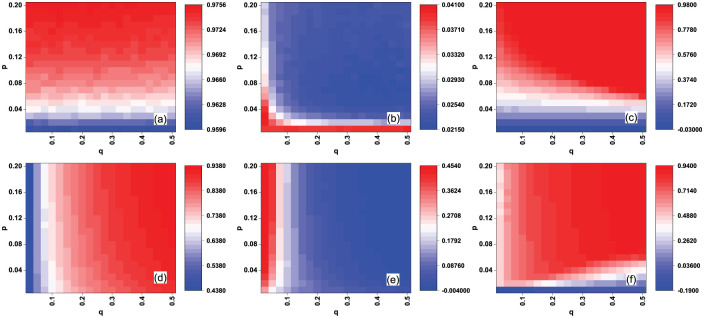
(Color online) The effectiveness of the EigenRank algorithm under malicious rating attack for the MovieLens data set: (a) the AUC values *AUC*_*e*_; (b-c) Δ*AUC*_*e*_, the comparison of *AUC*_*e*_ between EigenRank algorithm and IBeta method (b), Vote Aggregation method (c); (d) the recall *R*_*c*_(*L*); (e-f) Δ*R*_*c*_(*L*), the comparison of recall between EigenRank algorithm and IBeta method (e), Vote Aggregation method (f). The parameters *q* and *p* denote the ratio and the activity of spammers, respectively. *q* ranges from 0.025 to 0.5 in increments of 0.025 and *p* ranges from 0.01 to 0.2 in increments of 0.01. One observes that both the *AUC*_*e*_ and *R*_*c*_(*L*) of the EigenRank algorithm exceed their counterparts in the IBeta method and Vote Aggregation method for different (*p*, *q*).

Fig 2(a) and 2(d) shows the AUC values *AUC*_*e*_ and recall *R*_*c*_(*L*) of the EigenRank algorithm with different (*p*, *q*) in the random rating attack case for the MovieLens data set, and Fig 2(b), 2(c), 2(e) and 2(f) shows Δ*AUC*_*e*_ and Δ*R*_*c*_(*L*), the comparison of AUC values and recall between the EigenRank algorithm and IBeta method/Vote Aggregation method. From Fig 2(a) and 2(d) one sees that *AUC*_*e*_ of the EigenRank algorithm are mainly determined by *p*, the activity of spammers, while *R*_*c*_(*L*) depends mainly on *q*, the ratio of spammers. Fig 2(b), 2(c), 2(e) and 2(f) reveals that Δ*AUC*_*e*_ and Δ*R*_*c*_(*L*) are all positive values, implying that the AUC value of the EigenRank algorithm is larger than that of the IBeta method and Vote Aggregation method for each (p, q), and so it is with recall Rc(L). For instance, AUCe = 0.977673, ΔAUCe = 0.05542 (compared to IBeta method) and *R*_*c*_(*L*) = 0.89777, Δ*R*_*c*_(*L*) = 0.127881 (compared to IBeta method) when (*p*, *q*) = (0.1, 0.3). The effectiveness of the EigenRank algorithm with different (*p*, *q*) in the malicious rating attack case for the MovieLens data set are shown in Fig 3. The results are similar to those in Fig 2.

**Fig 4:**
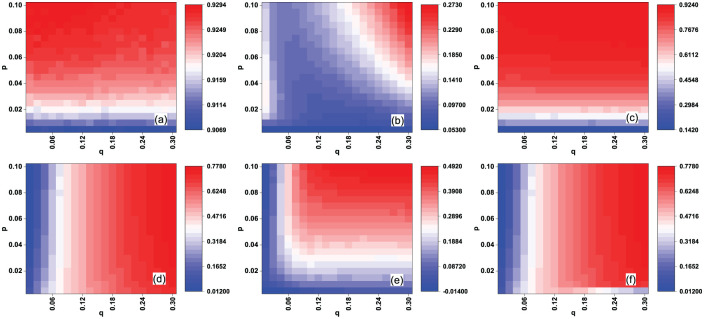
(Color online) The effectiveness of the EigenRank algorithm under random rating attack for the Netflix data set: (a) the AUC values *AUC*_*e*_; (b-c) Δ*AUC*_*e*_, the comparison of *AUC*_*e*_ between EigenRank algorithm and IBeta method (b), Vote Aggregation method (c); (d) the recall *R*_*c*_(*L*); (e-f) Δ*R*_*c*_(*L*), the comparison of recall between EigenRank algorithm and IBeta method (e), Vote Aggregation method (f). The parameters *q* and *p* denote the ratio and the activity of spammers, respectively and *L* = *q*|*U*|. *q* ranges from 0.015 to 0.3 in increments of 0.015 and *p* ranges from 0.005 to 0.1 in increments of 0.005. One observes that both the *AUC*_*e*_ and *R*_*c*_(*L*) of the EigenRank algorithm exceed those of the IBeta method and Vote Aggregation method for different (*p*, *q*).

**Fig 5:**
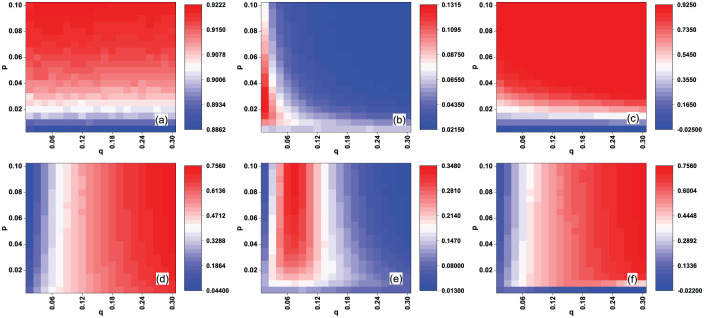
(Color online) The effectiveness of the EigenRank algorithm under malicious rating attack for the Netflix data set: (a) the AUC values *AUC*_*e*_; (b-c) Δ*AUC*_*e*_, the comparison of *AUC*_*e*_ between EigenRank algorithm and IBeta method (b), Vote Aggregation method (c); (d) the recall *R*_*c*_(*L*); (e-f) Δ*R*_*c*_(*L*), the comparison of recall between EigenRank algorithm and IBeta method (e), Vote Aggregation method (f). The parameters *q* and *p* denote the ratio and the activity of spammers, respectively and *L* = *q*|*U*|. *q* ranges from 0.015 to 0.3 in increments of 0.015 and *p* ranges from 0.005 to 0.1 in increments of 0.005. It can be seen that both the *AUC*_*e*_ and *R*_*c*_(*L*) of the EigenRank algorithm are larger than their counterparts in the IBeta method and Vote Aggregation method for different (*p*, *q*).

For the Netflix data set, Figs 4 and 5 show the effectiveness of the EigenRank algorithm in the random and malicious rating attack cases, respectively. One also observes that both the AUC values and recall of the EigenRank algorithm are larger than their counterparts in the IBeta method and Vote Aggregation method with different pair (*p*, *q*) under the random/malicious rating attack case. For example, *AUC*_*e*_ = 0.908, Δ*AUC*_*e*_ = 0.144 (compared to Vote Aggregation method) and *R*_*c*_(*L*) = 0.724, Δ*R*_*c*_(*L*) = 0.274 (compared to Vote Aggregation method) when (*p*, *q*) = (0.005, 0.3) in Fig 4; *AUC*_*e*_ = 0.915, Δ*AUC*_*e*_ = 0.024 (compared to IBeta method) and *R*_*c*_(*L*) = 0.658, Δ*R*_*c*_(*L*) = 0.081 (compared to IBeta method) when (*p*, *q*) = (0.05, 0.21) in Fig 5; The results for empirical networks (Figs 2–5) indicate that the EigenRank algorithm is of better accuracy and robustness than IBeta method and Vote Aggregation method in the rating attack cases.

## Conclusion and discussions

In this paper we present an iterative reputation and quality ranking algorithm via the eigenvector, called the EigenRank algorithm, where the user reputation and object quality are interdependent and the user reputation converges to a unique stationary ranking (the eigenvector corresponding to the largest eigenvalue of a certain matrix). In addition, we prove the convergence of EigenRank algorithm, and analyse its speed of convergence. Moreover, we explore the ranking performance of the EigenRank algorithm for the synthetic and empirical networks compared with the IBeta method and Vote Aggregation method. For the synthetic networks, the results indicate that the AUC values and Kendall’s *τ* of the EigenRank algorithm exceed the ones obtained from the IBeta method and Vote Aggregation method with different proportions of random/malicious ratings. The results for the empirical networks show that the EigenRank algorithm is of better accuracy and robustness than the IBeta method and Vote Aggregation method in the random and malicious rating attack cases. This work provides a ranking without uncertainty for the user reputation identification.

In future, we will examine the convergence of other reputation ranking algorithms. Moreover, the rating time is an important attribute of ratings, and presenting new reputation ranking method considering time factor is worthy of further investigation. Consequently, we will concentrate on searching for more accurate and robust reputation ranking algorithms which are absured to estimate the user reputation.

## Supporting information

S1 Appendix (PDF)
