# Supplementary material for: A converging reputation ranking iteration method via the eigenvector
Source: PLoS One. 2022 Oct 3;17(10):e0274567. doi: 10.1371/journal.pone.0274567 (PMC9529115; doi:10.1371/journal.pone.0274567)
Supplement: S1 Appendix — (PDF) [file pone.0274567.s001.pdf]

SUPPLEMENTARY INFORMATION
A converging reputation ranking iteration method via the eigenvector

Xiao-Lu Liu[1]*, Chong Zhao[2]*

[1]School of Management Science and Engineering, Shandong University of Finance and Economics, Jinan 250014, PR China;
[2]School of Mathematics, Shandong University, Jinan 250100, PR China

We prove the convergence of EigenRank algorithm, and analyse the speed of convergence. Meanwhile, we investigate the time complexity of the EigenRank algorithm.

1 Proof of convergence

As a consequence of Eq. (9) in the main text,

$$\mathbf{D}^{\frac{1}{4}}\mathbf{R} = c_1 c_2 \mathbf{D}^{-\frac{1}{4}}\mathbf{B}\mathbf{K}^{-\frac{1}{2}}\mathbf{B}^{\mathbf{T}}\mathbf{D}^{-\frac{1}{4}}\mathbf{D}^{\frac{1}{4}}\mathbf{R}. \tag{1}$$

Denote by $\tilde{\mathbf{R}} = \mathbf{D}^{\frac{1}{4}}\mathbf{R}, \tilde{\mathbf{B}} = \mathbf{D}^{-\frac{1}{4}}\mathbf{B}\mathbf{K}^{-\frac{1}{4}}, c = c_1c_2$, then (1) can be rewritten

$$\tilde{\mathbf{R}} = c\tilde{\mathbf{B}}\tilde{\mathbf{B}}^{\mathbf{T}}\tilde{\mathbf{R}}. \tag{2}$$

List the eigenvalues of $\tilde{\mathbf{B}}\tilde{\mathbf{B}}^{\mathbf{T}}$ in descending order as

$$\lambda_1 \geq \lambda_2 \geq \ldots \geq \lambda_k \geq 0,$$

where multiplicity is accounted. Choose an orthogonal matrix $\mathbf{G}$ such that

$$\mathbf{G}^{\mathbf{T}}\tilde{\mathbf{B}}\tilde{\mathbf{B}}^{\mathbf{T}}\mathbf{G} = \Lambda := \mathrm{diag}\{\lambda_1, \ldots, \lambda_k\}.$$

We make the assumption that λ_1 is the unique largest eigenvalue. Eq. (2) can be expressed as

$$\mathbf{x} = c\Lambda\mathbf{x}, \tag{3}$$

where $\mathbf{x} = \mathbf{G}^{\mathbf{T}}\tilde{\mathbf{R}}$.

The iteration can be expressed as

$$\mathbf{x}^{(n+1)} = \frac{\Lambda\mathbf{x}^{(n)}}{\|\mathbf{G}^{\mathbf{T}}\mathbf{D}^{-\frac{1}{4}}\mathbf{G}\boldsymbol{\Lambda}\mathbf{x}^{(\mathbf{n})}\|_2}, n = 0, 1, \ldots, \tag{4}$$

where $\mathbf{x}^{(n)}$ is the $\mathbf{x}$ after the n-th iteration step.

In the case $\mathbf{x}_1^{(0)} > 0$, Eq. (4) gives $\mathbf{x}_1^{(n)} > 0, \forall n$. Then we have

$$\frac{\mathbf{x}_i^{(n+1)}}{\mathbf{x}_1^{(n+1)}} = \frac{\lambda_i \mathbf{x}_i^{(n)}}{\lambda_1 \mathbf{x}_1^{(n)}}, \forall n \geq 0. \tag{5}$$

Consequently for $i \neq 1$ we have

$$\frac{\mathbf{x}_i^{(n)}}{\mathbf{x}_1^{(n)}} \leq \left(\frac{\lambda_i}{\lambda_1}\right)^n \frac{\mathbf{x}_i^{(0)}}{\mathbf{x}_1^{(0)}} \leq \eta^n \frac{\mathbf{x}_i^{(0)}}{\mathbf{x}_1^{(0)}}, \tag{6}$$

where by the assumption $\eta := \frac{\lambda_2}{\lambda_1} < 1$.

Denote by $e_1 = (1, 0, \ldots, 0)^T$. When $n \to \infty$, since $\frac{\mathbf{x}_i^{(n)}}{||\mathbf{x}^{(n)}||_2} \leq \frac{\mathbf{x}_i^{(n)}}{\mathbf{x}_1^{(n)}}$ decays to 0 for $i \neq 1$ and

$$||\mathbf{R}^{(n)}||_2 = ||\mathbf{D}^{-\frac{1}{4}}\mathbf{G}x^{(n)}||_2 \equiv 1, \forall n \geq 1, \tag{7}$$

we have $\|\mathbf{D}^{-\frac{1}{4}}\mathbf{G}x_1^{(n)}e_1\|_2 \to 1$, namely $x_1^{(n)} \to \frac{1}{\|\mathbf{D}^{-\frac{1}{4}}\mathbf{G}e_1\|_2}$. Therefore $\mathbf{x}^{(n)} \to \mathbf{x}^{(\infty)} := \frac{e_1}{\|\mathbf{D}^{-\frac{1}{4}}\mathbf{G}e_1\|_2}$, completing the proof of convergence.

2 Speed of convergence

Since $\mathbf{x}^{(n)} = \mathbf{G^T}\tilde{\mathbf{R}}^{(n)} = \mathbf{G^T}\mathbf{D}^{\frac{1}{4}}\mathbf{R}^{(n)}$ and $||\mathbf{R}^{(n)}||_2 \equiv 1$, it holds for all $n \geq 1$ that $||\mathbf{x}^{(n)}||_2 \leq ||\mathbf{D}^{\frac{1}{4}}||$. By formulae (6, 7) we have

$$\begin{aligned}
\big|||\mathbf{D}^{-\frac{1}{4}}\mathbf{G}x_1^{(n)}e_1||_2 - 1\big| &= \big|||\mathbf{D}^{-\frac{1}{4}}\mathbf{G}x_1^{(n)}e_1||_2 - ||\mathbf{D}^{-\frac{1}{4}}\mathbf{G}x^{(n)}||_2\big| \\
&\leq ||\mathbf{D}^{-\frac{1}{4}}\mathbf{G}(x^{(n)} - x_1^{(n)}e_1)||_2 \\
&\leq ||\mathbf{D}^{-\frac{1}{4}}|| \cdot ||x^{(n)} - x_1^{(n)}e_1||_2 \\
&\leq \eta^n||\mathbf{D}^{-\frac{1}{4}}|| \cdot ||x^{(0)} - x_1^{(0)}e_1||_2 \cdot \frac{x_1^{(n)}}{x_1^{(0)}} \\
&\leq \eta^n||\mathbf{D}^{-\frac{1}{4}}|| \cdot ||\mathbf{D}^{\frac{1}{4}}|| \cdot \frac{||x^{(0)}||_2}{x_1^{(0)}}.
\end{aligned} \tag{8}$$

It follows from these inequalities and Eq. (7) that

$$\begin{aligned}
||\mathbf{R}^{(n)} - \mathbf{R}^{(\infty)}||_2^2 &= 2 - 2\langle \mathbf{R}^{(n)}, \mathbf{R}^{(\infty)}\rangle \\
&= 2 - 2\langle \mathbf{D}^{-\frac{1}{4}}\mathbf{G}x^{(n)}, \mathbf{D}^{-\frac{1}{4}}\mathbf{G}x^{(\infty)}\rangle \\
&= 2 - 2\langle \mathbf{D}^{-\frac{1}{4}}\mathbf{G}x_1^{(n)}e_1, \mathbf{D}^{-\frac{1}{4}}\mathbf{G}x^{(\infty)}\rangle \\
&\quad -2\langle \mathbf{D}^{-\frac{1}{4}}\mathbf{G}(x^{(n)} - x_1^{(n)}e_1), \mathbf{D}^{-\frac{1}{4}}\mathbf{G}x^{(\infty)}\rangle \\
&= 2 - 2\langle \mathbf{D}^{-\frac{1}{4}}\mathbf{G}x_1^{(n)}e_1, \frac{\mathbf{D}^{-\frac{1}{4}}\mathbf{G}e_1}{\|\mathbf{D}^{-\frac{1}{4}}\mathbf{G}e_1\|_2}\rangle \\
&\quad -2\langle \mathbf{D}^{-\frac{1}{4}}\mathbf{G}(x^{(n)} - x_1^{(n)}e_1), \mathbf{D}^{-\frac{1}{4}}\mathbf{G}x^{(\infty)}\rangle \\
&\leq 2 - 2||\mathbf{D}^{-\frac{1}{4}}\mathbf{G}x_1^{(n)}e_1||_2 + 2||\mathbf{D}^{-\frac{1}{4}}\mathbf{G}(x^{(n)} - x_1^{(n)}e_1)||_2 \\
&\leq 2\eta^n||\mathbf{D}^{-\frac{1}{4}}|| \cdot ||\mathbf{D}^{\frac{1}{4}}|| \cdot \frac{||x^{(0)}||_2}{x_1^{(0)}} + 2\eta^n||\mathbf{D}^{-\frac{1}{4}}|| \cdot ||\mathbf{D}^{\frac{1}{4}}|| \cdot \frac{||x^{(0)}||_2}{x_1^{(0)}} \\
&= 4\eta^n||\mathbf{D}^{-\frac{1}{4}}|| \cdot ||\mathbf{D}^{\frac{1}{4}}|| \cdot \frac{||x^{(0)}||_2}{x_1^{(0)}}.
\end{aligned} \tag{9}$$

As a consequence

$$\begin{aligned}
||\mathbf{R}^{(n)} - \mathbf{R}^{(n-1)}||^2 &\leq 2||\mathbf{R}^{(n)} - \mathbf{R}^{(\infty)}||^2 + 2||\mathbf{R}^{(n-1)} - \mathbf{R}^{(\infty)}||^2 \\
&\leq 16\eta^{n-1}||\mathbf{D}^{-\frac{1}{4}}|| \cdot ||\mathbf{D}^{\frac{1}{4}}|| \cdot \frac{||x^{(0)}||_2}{x_1^{(0)}}.
\end{aligned} \tag{10}$$

Therefore, in order to guarantee

$$||\mathbf{R}^{(n)} - \mathbf{R}^{(n-1)}||^2 \leq 16\eta^{n-1}||\mathbf{D}^{-\frac{1}{4}}|| \cdot ||\mathbf{D}^{\frac{1}{4}}|| \cdot \frac{||x^{(0)}||_2}{x_1^{(0)}} < \delta, \tag{11}$$

such that the iteration stops, it is enough to let $n > \log_\eta \frac{x_1^{(0)}\delta}{16||\mathbf{D}^{-\frac{1}{4}}||\cdot||\mathbf{D}^{\frac{1}{4}}||\cdot||x^{(0)}||_2} + 1$.

3 Time complexity

In each step of iteration, there are 4 matrix multiplication of total $2|U||O| + |U| + |O|$ times of number multiplication, one summation of $|U|$ squares, one square root and $|U|$ divisions. Therefore each step is of time complexity $O(|U||O|)$. Since the iteration stops in no more than $[\log_\eta \frac{x_1^{(0)}\delta}{16||\mathbf{D}^{-\frac{1}{4}}||\cdot||\mathbf{D}^{\frac{1}{4}}||\cdot||x^{(0)}||_2}]+3$ steps, the time complexity of the iteration process in the EigenRank algorithm is not beyond $O(|U||O|\log_\eta \delta)$.

In the normalization process of the rating matrix, to find the extreme ratings r_i^1 and r_i^2 for all the users i, $2|E|$ times of comparisons are needed. Then the normalization via formula (**??**) contains $|E|$ times of division. Therefore the time complexity of the normalization process is $O(|E|)$.

Sum up, the total complexity of the EigenRank algorithm is $O(|U||O|\log_\eta \delta)$.
